# Long-Term Prevalence and Risk Factors of Musculoskeletal Disorders among the Schoolteachers in Hail, Saudi Arabia: A Cross-Sectional Study

**DOI:** 10.1155/2022/3610196

**Published:** 2022-03-18

**Authors:** Omar W. Althomali

**Affiliations:** Department of Physiotherapy, College of Applied Medical Sciences, University of Hail, Hail, Saudi Arabia

## Abstract

**Background:**

Musculoskeletal disorders (MSDs) are affecting up to 95% of teachers and are considered a primary occupational health hazard leading to absenteeism, early retirement, and lower quality of life and teaching quality.

**Aim:**

The current study is aimed at exploring the prevalence and risk factors of MSDs among the schoolteachers in Hail, Saudi Arabia.

**Methods:**

A cross-sectional study was conducted online among the teachers in Hail City using the Nordic Musculoskeletal Questionnaire. Teachers were randomly selected from randomly selected high schools. Teachers with at least 1 year experience were recruited for the study. Descriptive statistics, Cochran's *Q* test, and binominal regression were used to investigate the prevalence of MSDs among such teachers and to determine if the percentage of MSDs differed by anatomical region and risk factor (one hundred forty-five males and 106 females filled out the questionnaires).

**Results:**

The prevalence of MSDs in the last 12 months was 93.63% (235 of 251 teachers). Interestingly, 91% of the affected participants (214 of 235 teachers) complained of MSDs in more than one anatomical region. The most affected site was the lower back (183 of 251 teachers, 72.91%), followed by the shoulders (168 of 251 teachers, 66.93%), and the least affected sites were the elbows (45 of 251 teachers, 17.93%). The females showed a higher prevalence of MSDs than the males. Only gender was a significant risk factor for shoulder and neck MSDs (*p* < 0.02).

**Conclusion:**

Overall, the findings of the current study suggest a high prevalence of MSDs among teachers, especially in the lower back and shoulders. The affected teachers should learn more about biomechanics and ergonomics and should engage in exercise to improve their health. Future studies should focus on identifying the biomechanical and ergonomic risk factors of MSDs and on designing MSD prevention programmes to reduce the burden of MSDs.

## 1. Introduction

Musculoskeletal disorders (MSDs) have been identified as among the most important and common health problems in occupational populations, with several socioeconomic implications [1]. MSDs include a wide range of degenerative and inflammatory conditions affecting the joints, muscles, ligaments, tendons, bones, nerves, and blood system [2, 3]. They lead to reduced productivity due to increased absenteeism, sick leave, and early retirement, and they are costly to treat [2, 4]. In addition, MSDs have been identified as common causes of work discontinuance to seek medical treatment.

Work is essential for most people [5, 6]. One of the most important sectors in any country is the education system [7]. Teachers play an important role in the functioning of the education system and in improving the quality of the education process [8, 9]. However, the work of teachers, such as using computers and reading, and checking and scoring quizzes and assignments, often involves spending a significant amount of time in a head-down posture. Teachers also perform various tasks requiring sustained mechanical loads, with consistent and repeatable trunk flexion. Moreover, teaching is sometimes carried out in unsuitable environments, with long periods of standing [6, 10]. All these factors may increase teachers' risk of developing MSDs, which can lower their quality of life.

It has been anticipated that 39–95% of teachers are affected by MSDs during their career [8, 11]. In terms of body region affected by MSDs, teachers appear to have a greater risk of developing back, neck, and upper-limb MSDs [2, 9, 12]. As suggested by the literature, however, MSDs have multiple risk factors that may add to teachers' risk of developing them, such as female gender, sleeping disturbance, and smoking [8, 13, 14].

In Saudi Arabia, previous studies have demonstrated that 62.5–91% of teachers have MSDs [15–21]. In particular, such studies have demonstrated that the prevalence of back pain is 38.1–74.4%, that of shoulder pain is 20.6–57.5%, and that of neck pain is 42.3–58%. Interestingly, the prevalence of leg pain was found to be up to 58%, and it was found that pain affected the work at school by 46.1% [19]. Darwish and Al-Zuhair stated that the trends of increased prevalence of MSDs and association of several body regions with MSDs in Saudi Arabia in 2013 were similar to those across the globe.

However, the research in the aforementioned field is lacking. Only a few studies have identified the prevalence and risk factors of MSDs among teachers, and some of them focused on one gender [16–19], excluded many details regarding anatomical sites of MSDs [20, 21] or did not specify the gender [15]. To date, no single study has investigated the long-term prevalence of MSDs in both male and female teachers and their risk factors. If prevention and control measures are not planned, the problem may worsen and the number of teachers with disabilities may increase, which will increase teachers' health expenses. Thus, the current study investigated the prevalence and risk factors of MSDs among both male and female teachers.

## 2. Materials and Methods

A cross-sectional observational study was carried out among secondary schoolteachers in Hail, Saudi Arabia. To be included in the study, one had to be a teacher with at least 1 year teaching experience in a government school. Both genders were included in the study. Teachers with a malignant tumour, a physical disability, a previous surgery or an inflammatory joint disease, or who were pregnant, already retired, or working in a private school were excluded from the study.

### 2.1. Sampling

The Raosoft sample size calculator (Raosoft, USA) was used to compute the required sample size for the study, with a 95% confidence interval (CI) at a 5% marginal error rate and a 70% response rate. The required sample size was found to be 244 participants. The participant recruitment for the current study involved two steps to reduce the risk of bias. In the first step, a random sample of schools was selected from among the 42 public schools in the Hail region (with a total of 995 teachers). The average number of students per school is 225. Most of the schools operate in the mornings. The schools were labelled with even and odd numbers, and those labelled with even numbers were selected, as a method of randomisation. Thus, 21 schools were selected from an earlier prepared list. The second step involved the random selection of teachers from the selected schools. The teachers labelled with even numbers in these selected schools were selected and recruited for the study. A total of 304 teachers were recruited, and 254 of them filled out and returned the questionnaire (83.55% response rate). Of the 254 accomplished questionnaires, three were excluded due to missing necessary data. A total of 251 accomplished questionnaires were thus included in the statistical analysis.

### 2.2. Questionnaire

The Arabic version of the Nordic Musculoskeletal Questionnaire (NMQ) was used to collect the required data regarding the prevalence of MSDs among the selected teachers in the selected schools [17]. NMQ is a reliable, valid, and responsive instrument [22, 23]. Both the Arabic and English versions of the questionnaire have been used in previous studies to investigate musculoskeletal symptoms in work-related health contexts [23]. NMQ is divided into three main parts. The first part pertains to any pain felt in any of nine body regions within the last 12 months. The second part pertains to any pain felt in any of the same nine body regions but within the last 7 days. The third part pertains to whether a disability occurred due to pain in any of the same nine body regions. The nine anatomical regions are the neck, shoulders, elbows, wrists/hands, upper back, lower back, hips/thighs/buttocks, knees, and ankles/feet. The respondents' answers came in the form of binary responses to some questions (yes and no) and in the form of multinominal responses to other questions (no, right side, left side, and both sides).

### 2.3. Data Collection

The data were gathered using the online Google Form application. The first page of the form conveyed the study's purpose to the participants and sought their consent to participate in the study. The second page asked for the participants' demographic characteristics (age, gender, smoking habit, number of sleeping hours per day, length of job experience, number of working hours per day, and marital status). After the form's creation, it was first sent to peers for checking. Then, it was piloted by the researcher on approximately 10 participants, to check for clarity. The survey link was shared with the participants through their WhatsApp accounts and via emails. Thereafter, reminders were sent to the participants (for them to accomplish the survey questionnaire) to improve the response rate.

### 2.4. Ethical Considerations

The current study was approved by the ethical committee of Hail University under ethical approval number H-2020-007. The study was conducted from February to April 2020.

### 2.5. Statistical Analysis

The collected data were exported from Google Form to a Microsoft Excel (version 16.33) spreadsheet then to SPSS version 25 for analysis (SPSS, Inc., Chicago, IL, USA). All the multinominal data were transformed into binary data (yes or no), as follows: “yes” if the answer was on the left side, right side, or both sides, and “no” was left as “no.” The data related to the pain felt within the last 12 months were analysed as they could reflect a longer pain duration and could represent a better time range than the data related to the pain felt within only the last 7 days. Moreover, the data covering the last 12 months showed that all the regions had higher MSD prevalence than in the last 7 days, ranging from 5.58% to 13.55%. Percentage and frequency analyses were used to present the data, and Microsoft Excel was used to construct the bar charts. The data were also split and presented by gender. Cochran's *Q* test was used to determine if there were differences in the pain felt in the body regions within the last 12 months. To predict the demographic characteristics that could influence the top three common sites of pain within the last 12 months, binominal regression was used. Each variable was entered into a separate regression model. Statistical significance was set at below 0.05.

## 3. Results

The current study had 251 participants, 145 (57.8%) of whom were males and 106 (42.2%) females. As can be seen in Table 1, most of the participants were married (85.7%) and nonsmokers (78.1%). Approximately half (47.8%) were 36–45 years old. Moreover, 53% claimed to sleep 7–8 hours daily. Lastly, most (36.3%) had a job experience of over 20 years.

**Figure 1:**
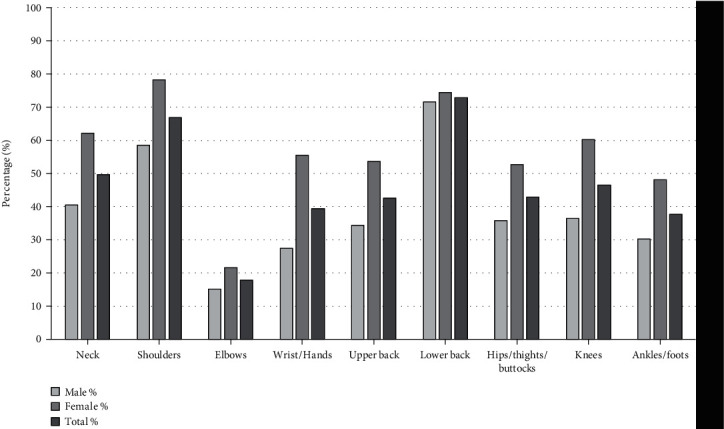
Percentages of musculoskeletal disorders in the last 12 months among the teacher participants in this study.

Interestingly, 93.63% of the participants (235 of 251 teachers) developed MSDs within the last 12 months. The females showed a higher prevalence of MSDs (99.1%, 105 of 106 teachers) than the males (89.7%, 130 of 145 teachers). The Cochran's *Q* test results statistically differed by body region (*χ*^2^(8) = 263.65; *p* < 0.0005). The lower back was the most common site of MSDs (183, 72.91%), followed by the shoulders (168, 66.93%), neck (125, 49.8%), knees (117, 46.61%), hips/thighs/buttocks (108, 43.03%), upper back (107, 42.63%), wrists/hands (99, 39.44%), ankles/feet (95, 37.85%), and elbows (45, 17.93%). Among the males, the lower back was the most common site of MSDs (104, 71.72%), followed by the shoulders (85, 58.62%), and the least common sites of MSDs were the elbows (22, 15.17%). Among the females, the most common sites of MSDs were the shoulders (83, 78.3%), followed by the lower back (79, 74.53%), and the least common sites of MSDs were also the elbows (23, 21.7%) (Tables 2 and 3, Figure 1).

Binominal logistic regression was performed to investigate the relationships between seven demographic characteristics (gender, age, smoking habit, length of job experience, number of working hours per day, number of sleeping hours per day, and marital status) and the relationships between lower back, shoulder, and neck MSDs. The model explanation and the correct classification of the models were for the lower back (1.1%, 72.9%), shoulders (8%, 64.1%), and neck (7.2%, 58.8%). Of the seven demographic characteristics, only gender showed significant results (*p* < 0.02) in two regions (shoulders and neck). The females had 2.46- and 2.51-fold higher risks of developing MSDs in the shoulders and neck, respectively (Table 4).

## 4. Discussion

The current study investigated the prevalence and risk factors of MSDs among schoolteachers in Hail, Saudi Arabia. Teaching is considered one of the professions most affected by MSDs [2, 12, 17, 24]. Despite this, however, few studies have investigated the long-term prevalence of MSDs among schoolteachers [17–21].

The results of the current study show that MSDs are highly prevalent among the teachers in Hail, Saudi Arabia. More than 90% of the participants developed MSDs within the last 12 months. Interestingly, 8.4% complained of MSDs in a single body region while 85.2% complained of MSDs in more than one body region. The lower back, shoulders, and neck were the most common MSD sites, followed by the knees. Surprisingly, the females demonstrated a higher prevalence of MSDs than the males in all the body regions, almost twofold in some body regions, such as the wrists/hands. This is consistent with the finding of previous studies involving the teachers in Saudi Arabia: that the prevalence of MSDs among such teachers is 62.5–91% [15–21].

MSD is considered one of the main occupational health risks in working environments. Furthermore, MSDs are considered major causes of absenteeism, lower work quality, and early retirement of teachers due to the limited physical and professional functioning of the affected individuals, which in turn affects the economy. Other studies in China and Turkey have found 66.7% and 60.3% MSD prevalence rates among the teachers in such countries, respectively [8, 25]. The prevalence rate in Hail, Saudi Arabia, that was found in the current study is higher than those found in other studies in China (31.8%) [5] and Turkey (28.0%) (13) and is close to that found in other studies in Taiwan (86%) [26], China (77%) [27], and Saudi Arabia (79.17%) [17]. The difference in percentages may be due to certain factors, such as differences in populations and duration of MSD.

The higher prevalence of MSDs among teachers is highlighted by other studies in different countries. Interestingly, a systematic review in 2011 reported that among teachers, the prevalence of MSDs lies between 39% and 95% [8]. This is consistent with the finding of the current study: that the prevalence of MSDs among teachers is 93.63%. It is also consistent with the findings of previous studies conducted in Saudi Arabia among other populations: physiotherapists [28] and dentists [29].

The results of the current study also indicate that the lower back (72.91%) and shoulders (66.93%) are the body regions most affected by MSDs among teachers. Similar results were found in previous studies on the prevalence of MSDs among Saudi teachers, indicating a lower back MSD prevalence of up to 74.4% [15–21]. Interestingly, in previous studies in Saudi Arabia, the prevalence of shoulder pain among teachers was found to range from 20.6% to 57.5%, slightly lower than in the current study [15–21]. This may be explained by the differences in the study samples' demographic characteristics, in the questionnaire used, and in the region from which the study data were collected. Moreover, the shoulder pain prevalence rates obtained in current study in Saudi Arabia are in line with those obtained in other studies conducted in China [27], Turkey [30], Brazil [24], and Malaysia [24], which showed that the lower back is the body region most affected by MSDs. Moreover, the lower back was also listed as the body region most affected by MSDs among other professionals, such as physiotherapists (68.8%) [28] and dentists (79%) [29, 31].

The high prevalence of lower back pain may indicate the overuse and overloading of the spine. Teachers are prone to bad posture and body mechanics while sitting and standing for a long time. A 2007 systematic review noted that prolonged sitting with body twisting and awkward positioning increases the risk of lower back pain [32]. The high prevalence of shoulder pain may be due to computer use or writing on the whiteboard with the shoulders elevated. This position leads to compression of the humeral head against the coracoacromial arch, resulting in reduced blood circulation. There is evidence that repeated overhead activities contribute to overuse injuries and function impairment [33].

Among the variables that were tested to investigate the risk factors of MSD, only gender was found to be a significant risk factor, particularly for shoulder and neck MSDs but not for lower back MSDs. Being female increased the risk of developing an MSD 2.46- and 2.51-fold in the shoulders and neck, respectively. Previous systematic reviews have demonstrated that gender is considered a risk factor depending on the body region, which supports the findings of the current study [34]. However, bad posture and ergonomic or psychological factors may be even more important risk factors than those considered in the current study [34]. The high prevalence of MSDs among teachers could be largely due to their bad posture and prolonged standing, but this is only based on speculation and needs further investigation. Horng et al. found that inappropriate body mechanics such as twisting and leaning forward and long periods of standing and bad posture while teaching and using a computer are correlated with MSDs. This may be explained by the increase in the external moment on the joint, which overloads it and thereby causes pain.

## 5. Limitations and Future Studies

As with any research, this study had several limitations. First, a limited number of risk factors were investigated. Although other biomechanical factors may be more important risk factors for MSDs, adding more variables to those considered in the current study would have increased the time required to fill out the questionnaire and could have thus reduced the response rate. Therefore, future studies should focus on other possible risk factors and how to address them to reduce the prevalence of MSDs and their impact on individuals and on the workplace and economy.

Second, the current study used a questionnaire survey to investigate the prevalence of MSDs among teachers. While the results of the use of this method may be affected by recall bias, especially with the long duration of pain experience considered (12 months), it has the advantage of obtaining the required data within a short time. Future studies may combine a questionnaire survey with small-sample interviews to enhance the study method used and to thus obtain better results. Future studies should design an intervention on the basis of the current prevalence rate of MSDs among teachers to address such problem and to monitor any decrease in the rate.

## 6. Conclusion

The current study revealed that MSDs, especially lower back and shoulder MSDs, are prevalent among schoolteachers. Furthermore, there is a higher prevalence of MSDs among females than among males in all the MSD anatomical sites. This may be explained by teachers' low awareness of ergonomics and of the biomechanics of bad posture. Therefore, MSD prevention programmes should focus on these factors to reduce the negative effects of MSDs on individuals and the economy, such as absenteeism and frequent clinical consultation. In addition, the Ministry of Education should focus on reducing the prevalence of MSDs among teachers to minimise their effect on teachers' performance and quality of teaching. The findings of the current study can serve as the bases for future research focusing on biomechanics and the ergonomic and psychological risk factors of MSDs.

## Figures and Tables

**Table 1:** Demographic characteristics of the sample (*n* = 251).

Characteristics	Categories	*N*	%	Characteristics	Categories	*n*	%
Gender	Male	145	57.8	Smoking habit	Nonsmokers	196	78.1
	Female	106	42.2		Smokers	55	21.9

Age (years)	26–30	30	12.0	Number of sleeping hours per day	2–4	10	4.0
	31–35	23	9.2		5–6	79	31.5
	36–40	59	23.5		7–8	133	53.0
	41–45	61	24.3		9–10	26	10.4
	46–50	50	19.9		11–12	1	0.4
	>50	28	11.2		>12	2	0.8

Number of working hours per day	2–4	15	6.0	Length of job experience (years)	1–5	22	8.8
	5–6	95	37.8		6–10	43	17.1
	7–8	120	47.8		11–15	51	20.3
	9–10	14	5.6		16–20	44	17.5
	>10	7	2.8		>20	91	36.3

Marital status	Never married	28	11.2				
	Married	215	85.7
	Widowed	6	2.4
	Divorced	2	0.8

**Table 2:** Prevalence and distribution of musculoskeletal disorders (*n* = 251).

Response	Number of participants (%)
Musculoskeletal disorders	
Yes	235 (93.63)
No	16 (6.37)

Gender	
Male	130 (89.7)
Female	105 (99.1)

Body region involved	
No	16 (6.4)
Single region	21 (8.4)
Two regions	31 (12.4)
Three regions	30 (12)
Four regions	55 (21.9)
Five regions	22 (8.8)
Six regions	24 (9.6)
Seven regions	28 (11.2)
Eight regions	12 (4.8)
Nine regions	12 (4.8)

**Table 3:** Distribution of musculoskeletal disorders in body regions (*n* = 251).

Body region	Total number	%	Number of males	%	Number of females	%
Neck	125	49.80	59	40.69	66	62.26
Shoulders	168	66.93	85	58.62	83	78.30
Elbows	45	17.93	22	15.17	23	21.70
Wrist/hands	99	39.44	40	27.59	59	55.66
Upper back	107	42.63	50	34.48	57	53.77
Lower back	183	72.91	104	71.72	79	74.53
Hips/thighs/buttocks	108	43.03	52	35.86	56	52.83
Knees	117	46.61	53	36.55	64	60.38
Ankles/feet	95	37.85	44	30.34	51	48.11

**Table 4:** Binominal regression results for lower back, shoulder, and neck musculoskeletal disorders with demographic characteristics.

Variable	*B*	SE	Wald	df	Sig.	Exp(*B*)	95% CI for Exp(*B*)	
							Lower	Upper
Lower back								
Gender (female)	0.108	0.316	0.117	1	0.732	1.114	0.6	2.07
Age	-0.122	0.154	0.622	1	0.43	0.886	0.655	1.198
Smoking habit (yes)	-0.084	0.366	0.053	1	0.818	0.919	0.449	1.883
Length of job experience (years)	0.039	0.167	0.055	1	0.815	1.04	0.75	1.441
Number of sleeping hours per day	-0.171	0.185	0.851	1	0.356	0.843	0.586	1.212
Number of working hours per day	-0.017	0.179	0.009	1	0.926	0.984	0.692	1.398
Marital status	0.082	0.354	0.054	1	0.816	1.086	0.543	2.172
Constant	1.602	0.903	3.145	1	0.076	4.964		

Shoulders								
Gender (female)	0.9	0.314	8.217	1	**0.004**	2.459	1.329	4.548
Smoking habit (yes)	-0.149	0.341	0.19	1	0.663	0.862	0.442	1.681
Age	-0.057	0.148	0.151	1	0.697	0.944	0.707	1.261
Length of job experience (years)	0.217	0.161	1.823	1	0.177	1.242	0.907	1.703
Number of sleeping hours per day	-0.153	0.181	0.715	1	0.398	0.858	0.601	1.224
Number of working hours per day	-0.103	0.173	0.355	1	0.552	0.902	0.643	1.265
Marital status	-0.026	0.314	0.007	1	0.934	0.974	0.527	1.802
Constant	-0.34	0.846	0.161	1	0.688	0.712		

Neck								
Gender (female)	0.919	0.287	10.25	1	**0.001**	2.507	1.428	4.401
Age	-0.067	0.141	0.23	1	0.632	0.935	0.709	1.232
Smoking habit (yes)	0.161	0.336	0.229	1	0.632	1.175	0.608	2.271
Length of job experience (years)	-0.055	0.152	0.133	1	0.715	0.946	0.703	1.274
Number of sleeping hours per day	-0.123	0.171	0.518	1	0.472	0.884	0.633	1.236
Number of working hours per day	0.089	0.165	0.293	1	0.588	1.093	0.792	1.510
Marital status	0.118	0.307	0.147	1	0.702	1.125	0.616	2.053
Constant	-0.913	0.809	1.274	1	0.259	0.401		

Values indicated in boldface are significant.

## Data Availability

The data is available on request. Please contact Dr. Omar Althomali (o.althomali@uoh.edu.sa).
